# *FGFR2* Fusions in Pancreatobiliary and Ampullary Cancers: High Detection Rate in FFPE Specimens of Intrahepatic Cholangiocarcinoma with Limited RNA Yields Using Amplicon-Based NGS Assay

**DOI:** 10.3390/curroncol33090559

**Published:** 2026-09-15

**Authors:** Simon Guertin, Niloofar Sina, Ken Kron, Kenneth J. Craddock, Amy McCarty, David M. Hwang, Weei-Yuarn Huang

**Affiliations:** 1 Department of Clinical Pathology, Quebec University Hospital Center, Laval University, Quebec, QC G1R 2J6, Canada; simon.guertin@umontreal.ca; 2 Laboratory Medicine Program, Department of Anatomic al Pathology, University Health Network, University of Toronto, 200 Elizabeth St, Toronto, ON M5G 2C4, Canada; niloofar.sina@uhn.ca; 3 Department of Laboratory Medicine and Molecular Diagnostics, Sunnybrook Health Sciences Centre, University of Toronto, 2075 Bayview Avenue, North York, Toronto, ON M4N 3M5, Canada; ken.kron@sunnybrook.ca (K.K.); kenneth.craddock@sunnybrook.ca (K.J.C.); amy.mccarty@sri.utoronto.ca (A.M.); david.hwang@sunnybrook.ca (D.M.H.)

**Keywords:** intrahepatic cholangiocarcinoma, *FGFR2* fusion, amplicon-based NGS, RNA fusion testing, FFPE specimens, low input RNA, precision oncology

## Abstract

Fibroblast growth factor receptor 2 (FGFR2) fusions are identified in approximately 10–16% of patients with intrahepatic cholangiocarcinoma (iCCA) and represent actionable targets, with several FGFR inhibitors approved for clinical use. To evaluate the clinical utility and assay performance of an amplicon-based next-generation sequencing (NGS) approach in this setting, we assessed *FGFR2* fusion detection using the Oncomine Comprehensive Assay Plus (OCA-P). A total of 75 formalin-fixed paraffin-embedded (FFPE) specimens from biliary, pancreatic, and ampullary malignancies submitted for FGFR2 fusion testing between 1 March 2023 and 30 June 2024 were retrospectively analyzed, including 40 cases of iCCA. All iCCA specimens were successfully analyzed. *FGFR2* fusions were detected exclusively in iCCA, yielding a detection rate of 20%. RNA concentrations in iCCA specimens ranged from 0.02 ng/µL to 104 ng/µL; six cases (15%) exhibited yields below 1 ng/µL and 10 cases (25%) ranged between 1 and 2 ng/µL. Despite limited RNA input, OCA-P demonstrated robust performance, successfully identifying *FGFR2* fusions at concentrations below 2 ng/µL, levels typically considered suboptimal for partner-agnostic NGS approaches. These findings support the robustness and clinical utility of the OCA-P assay for reliable *FGFR2* fusion detection in iCCA, particularly in specimens with limited RNA input.

## Simple Summary

Gene fusions encompassing Fibroblast Growth Factor Receptor 2 (FGFR2) are important treatment targets in intrahepatic cholangiocarcinoma, but detecting them can be difficult in biopsy specimens that contain limited quantities of RNA. We evaluated a targeted sequencing approach for *FGFR2* fusion testing in 75 routine clinical biopsy samples, including 40 intrahepatic cholangiocarcinomas. All 40 cholangiocarcinomas were successfully analyzed, and *FGFR2* fusions were identified in eight cases (20%). Half of the positive cases had very low RNA concentrations. In one patient, the fusion was detected by our assay but not by a broader sequencing method, and this patient subsequently responded to targeted FGFR2 therapy. These findings support a targeted approach as a reliable method for *FGFR2* fusion detection in biopsy specimens when RNA quantity or quality is limited.

## 1. Introduction

Cholangiocarcinomas (CCA) are rare malignant neoplasms arising in the biliary epithelium that account for approximately 3% of all gastrointestinal cancers. Intrahepatic cholangiocarcinoma (iCCA) occurs proximal to bifurcation of the left and right hepatic ducts of the liver, whereas extrahepatic cholangiocarcinomas (eCCA) originate from the extrahepatic bile ducts. eCCA can be further subdivided into hilar cholangiocarcinoma (hCCA), occurring above the cystic duct, and distal cholangiocarcinoma (dCCA), arising below it [1,2]. The second most common primary hepatic malignancy after hepatocellular carcinoma is iCCA, and in recent decades, it has been diagnosed with increasing frequency [3].

Comprehensive genomic profiling using NGS has identified recurrent oncogenic alterations in genes including *ARID1A*, *BAP1*, *ERBB2*, *FGFR2*, *IDH1/2*, *KRAS*, and *TP53*. Molecular stratification has revealed that *BAP1*, *IDH1/2*, and *FGFR2* alterations are enriched in small-duct type iCCA, whereas *KRAS* and *TP53* alterations are more frequently observed in large-duct type tumors. The overall prognosis for small-duct iCCA remains poor, despite its association with improved survival rates relative to large duct disease. Currently, surgical resection is the only feasible option for a cure; however, most patients are unable to benefit due to their diseases presenting as unresectable or metastatic [4,5].

Recent advances in precision oncology have led to the development of targeted therapies, particularly FGFR inhibitors, which have demonstrated clinical efficacy and received regulatory approval for patients with unresectable or metastatic iCCA harboring *FGFR2* rearrangements [6,7,8,9,10,11].

*FGFR2* fusions are reported in approximately 10–16% of iCCA cases, with *BICC1* representing the most common fusion partner [6,7]. Notably, more than 100 *FGFR2* fusion partners have been described, the majority of which are rare or novel [11,12]. This diversity presents a significant analytical challenge for detection.

To address this heterogeneity, partner-agnostic RNA-based NGS approaches, including hybrid-capture and anchored multiplex PCR (AMP) methodologies, are widely recommended due to their ability to detect novel fusion partners. However, these approaches require relatively high-quality RNA and substantial input quantities. In contrast, amplicon-based NGS assays require lower RNA input and are more tolerant of degraded nucleic acid but are limited to predefined fusion targets [13,14,15,16,17,18]. Even with optimization, targeted panels with restricted fusion coverage may demonstrate reduced sensitivity, missing a substantial proportion of *FGFR2* rearrangements in proficiency studies [19].

A recent study using a partner-agnostic RNA-based NGS platform on resection specimens demonstrated near-complete analytical success rates, with an *FGFR2* fusion prevalence of 9.7%, using a minimum RNA input of 200 ng [19]. Notably, several novel fusion partners were identified, supporting the added value of partner-agnostic approaches in resected specimens.

In routine clinical practice, however, the majority of specimens submitted for molecular testing are biopsy samples obtained from patients with unresectable or metastatic disease. These samples frequently yield limited and degraded RNA, posing significant challenges for fusion detection.

In this study, we evaluated the performance of the OCA-P, an amplicon-based NGS assay, for *FGFR2* fusion detection in iCCA, with a particular focus on its applicability in low-input FFPE biopsy specimens. Our objective was to assess whether this approach can provide reliable and clinically actionable results in real-world diagnostic settings.

## 2. Materials and Methods

### 2.1. Case Selection

The study was conducted in concordance with the Declaration of Helsinki and was approved by the Institutional Review Board of Sunnybrook Health Sciences Center (REB#5386) on 30 June 2022. The requirement for written informed consent was waived due to the retrospective nature of the study. All cases submitted for *FGFR* fusion testing using formalin-fixed paraffined-embedded (FFPE) tissue were identified through a search of the next-generation sequencing (NGS) database at the Sunnybrook Health Sciences Centre molecular laboratory, covering the period from 1 March 2023, the start date for bile duct cancer testing, through 30 June 2024. For each case, associated pathology reports, clinical documentation, and radiology findings were reviewed to determine the anatomic site of tumor origin. Cases were classified as iCCA, hCCA, dCCA, gallbladder cancer (GC), ampullary adenocarcinoma, periampullary adenocarcinoma, or pancreatic adenocarcinoma. Ampullary, periampullary, and pancreatic adenocarcinomas were collectively categorized as non-biliary cancers.

Demographic information, including patient age and sex at the time of biopsy or surgery, was recorded. Additional data collected from the molecular laboratory database included specimen type, tumor cellularity, nucleic acid yield, and detected genomic alterations. Only DNA variants deemed clinically actionable were included in the analysis. Statistical analysis of categorical variables was performed using Fisher’s exact test, which is appropriate for categorical data when expected cell counts are small in 2 × 2 contingency tables [20].

The OCA-P validation cohort consists of 29 FFPE samples harboring 14 *NTRK*, 8 *ROS1*, 6 *ALK* fusions and 1 *MET* exon 14 skip. A commercial reference material, SeraSeq Fusion RNA Mix v4 (SeraCare, Milford, MA, USA, Cat # 0710-0497), was used to determine limits of detection (LOD) and run precision.

### 2.2. Nucleic Acid Extraction, Quantification, Targeted NGS Assays and Bioinformatic Analysis

FFPE tumor samples were macrodissected from 15 unstained sections (5 µm thickness) and subjected to nucleic acid extraction using the Promega Maxwell RSC system (Promega, Madison, WI, USA; Cat # AS4500). DNA and RNA were quantified using Qubit 4 (Thermo Fisher Scientific Inc., Waltham, MA, USA; Cat # Q33226) and Quantus platforms (Promega, Madison, WI, USA; Cat # E6150), respectively. Genomic alterations were assessed using the Oncomine Comprehensive Assay v3 (OCAv3; Thermo Fisher Scientific Inc., Waltham, MA, USA; Cat # A35805) for DNA-based variant detection and the Oncomine Comprehensive Assay Plus (OCA-P; Thermo Fisher Scientific Inc., Waltham, MA, USA; Cat # A49671) for RNA-based fusion analysis. Both assays are designed and optimized for FFPE tissue. The standard input requirements were 10 ng of DNA and 15 ng of RNA; for samples yielding less RNA, the maximum available RNA was used provided that DNA input criteria were met. OCAv3 DNA and OCA-P libraries were prepared on the Ion Chef (Thermo Fisher Scientific Inc., Waltham, MA, USA) using the AmpliSeq workflows, in which two DNA primer pools and two RNA primer pools are subjected to the standard 16-cycle DNA and 26-cycle RNA target-amplification program, respectively. The 26-cycle RNA program was applied routinely and was not selectively increased for low-input specimens. The Ion Chef automated library generation, equalization, and pooling, thereby reducing manual pipetting variation. Prepared libraries underwent automated template preparation and loading on Ion 540 chips, followed by sequencing on the Ion S5XL platform (Thermo Fisher Scientific Inc., Waltham, MA, USA).

Data analysis was conducted using Ion Reporter software (Thermo Fisher Scientific Inc., Waltham, MA, USA, version 5.18) with the extended filter chain against hg19, alongside custom modifications to enhance detection of *MET* exon 14 skipping events. Filtered-in calls were reviewed, including *FGFR2* exon tiling imbalance, if present. Fusion-supporting read counts and the percentage of total mapped fusion-panel reads were recorded. A cutoff of 0.25% fusion-supporting reads relative to total mapped panel reads and at least 200 fusion-supporting reads were required for a reportable call. For samples with 200–2000 supporting reads, orthogonal testing was performed for confirmation. Any *FGFR2* exon tiling imbalance call was regarded as a screening signal for a possible unrepresented fusion partner; positive imbalance prompted molecular-pathologist review and follow-up with orthogonal testing.

Sequencing runs were considered acceptable if quality control metrics—including mapped reads, on-target rates, sequencing depth, read length, uniformity, and MAPD scores—met manufacturer-recommended thresholds [21]. RNA-specific quality metrics included ≥500,000 mapped reads, adequate control signal in both primer pools (>100,000 reads each), mean read length >60 bp, and detection of at least four expression control targets. Samples not meeting one or more criteria were repeated, and a failed result was issued after two failed attempts. Each RNA library run included a no-template control, and a positive control was tested monthly. Run failures and monthly RNA success rates were logged and trended, with investigation of repeated control or instrument failures.

## 3. Results

### 3.1. Validation of OCA-P and Participation in External Proficiency Testing

Formal assay validation used 15 ng RNA per sample with OCA-P workflow w2.1 and the Oncomine Extended filter chain against hg19. Six pooled RNA validation libraries had a mean concentration of 130.3 pM (range, 94–158 pM). The validation evaluated 29 expected fusion variants such as *ALK1*, *NTRKs*, or *ROS1* fusions with no false-negative and no false-positive calls; within the tested validation set, reported sensitivity, specificity, and positive predictive value were each 100%. Reference RNA fusion materials run in duplicate across two runs demonstrated excellent precision on percentage of mapped reads across 18 different fusion variants present in the SaraSeq fusion RNA Mix.

External quality assurance (EQA)/proficiency testing was also employed for the assay using CAP (College of American Pathologist) and the Canadian Biomarker Quality Assurance (CBQA) program. No FGFR2 challenge specimen was present in the supplied CAP solid tumor RNA fusion EQA surveys. However, the laboratory correctly detected all five *FGFR3* fusion challenges in the supplied individual CAP reports: *FGFR3::BAIAP2L1* in RNA-A 2022, *FGFR3::TACC3* in RNA-A 2023, *FGFR3::BAIAP2L1* in RNA-B 2024, and both *FGFR3::BAIAP2L1* and *FGFR3::TACC3* in RNA-B 2025; all were graded “Good”.

In the 2024 Canadian Biomarker Quality Assurance (CBQA) educational *FGFR2* scheme, the laboratory correctly identified *FGFR2::SORBS3* and two *FGFR2::BICC1* samples. In the fourth sample, containing *FGFR2::STAU2*, the assay detected *FGFR2* expression imbalance and appropriately indicated that additional testing was required to identify the partner. Thus, three of four fusions were identified directly and the fourth generated a screen-positive imbalance signal.

### 3.2. Clinical Evaluation of OCA-P on Intrahepatic Cholangiocarcinoma

A total of 75 cases were tested for *FGFR2* fusions using the validated OCA-P between 1 March 2023 and 30 June 2024, at the molecular diagnostic laboratory of Sunnybrook Health Sciences Centre. The cohort included 40 male (53.3%) and 35 female (46.7%) patients, with ages ranging from 41 to 85 years (median and mean: 66 years).

**Figure 1:**
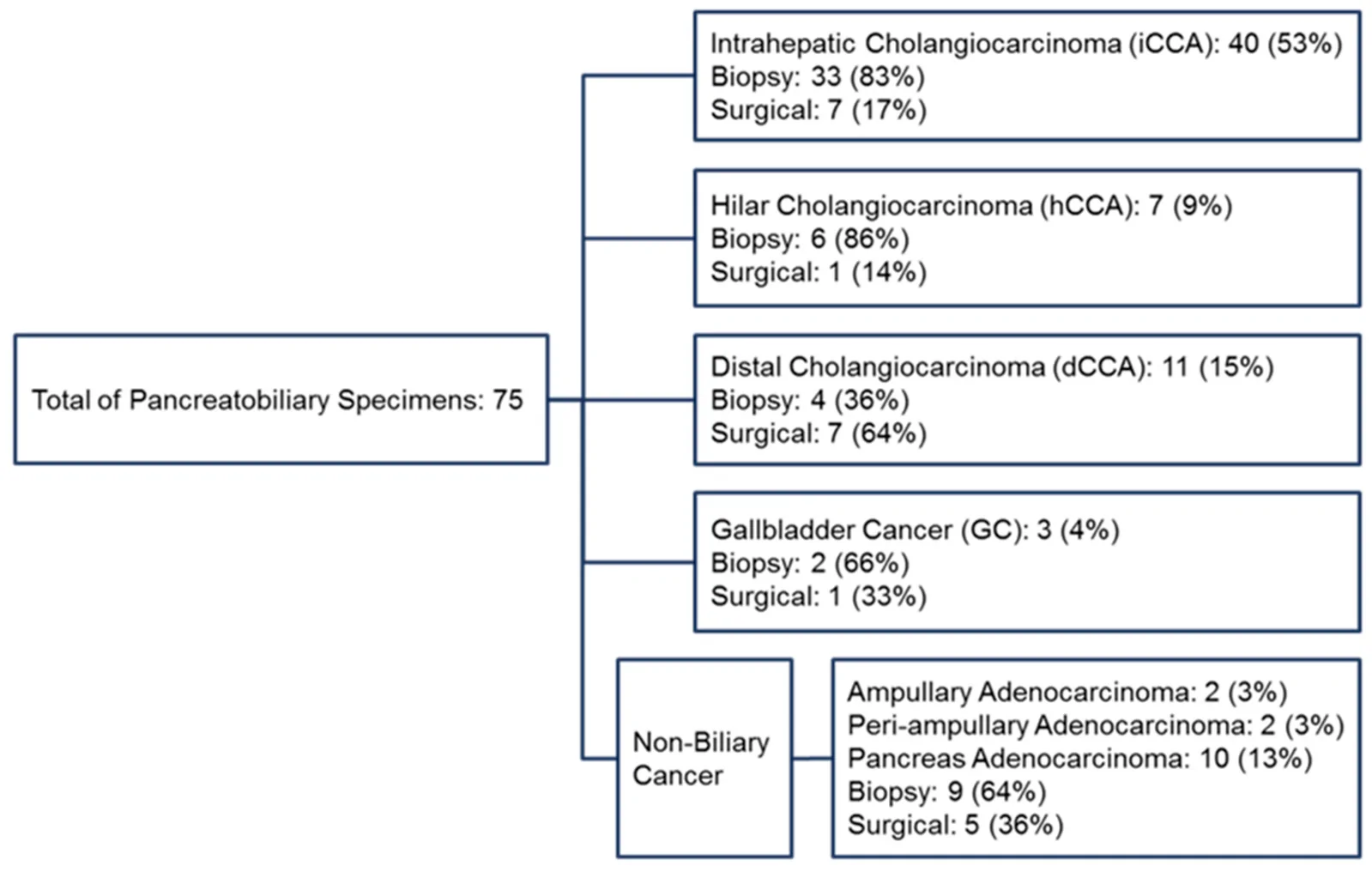
Classification of pancreatobiliary specimens tested for *FGFR2* fusion by OCA-P.

Of the 75 cases, 40 (53%) were classified as iCCA, comprising 33 biopsy and 7 surgical specimens. The anatomical classification of all tumors and the distribution of specimen types are summarized in Figure 1.

**Figure 2:**
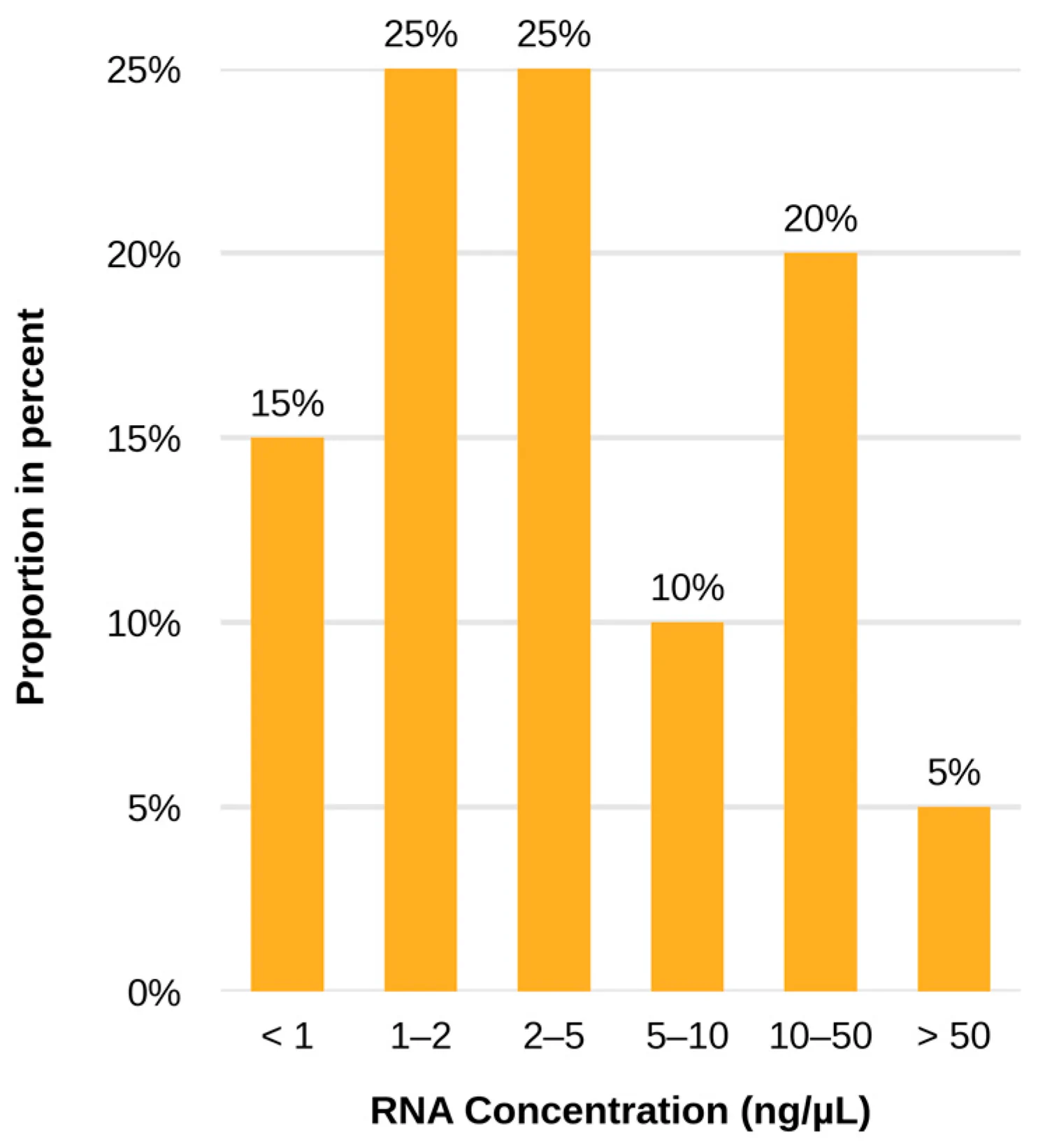
RNA concentration groups for the iCCA specimens tested by OCA-P.

RNA concentrations across all specimens, including iCCA, ranged from 0.02 ng/µL to 104 ng/µL. The corresponding total RNA input for sequencing of the specimen with yield of 0.02 ng/µL would be 0.3 ng only. Among iCCA cases, 6 (15%) had RNA yields below 1 ng/µL, and 10 (25%) had yields between 1 and 2 ng/µL. The distribution of RNA yields for iCCA cases is illustrated in Figure 2.

In a set of 614 unique samples comprising lung, bladder, thyroid and biliary tract tumors tested across 50 consecutive OCA-P runs, including the study samples, RNA yield in general was not associated with total mapped fusion-panel reads (Spearman ρ = −0.021; *p* = 0.6002), except at very low RNA concentrations (recorded at zero ng/µL concentrations). Median total mapped fusion-panel reads were 1,656,850 for samples with RNA concentration <1 ng/µL, 1,648,325 for samples with 1 to <2 ng/µL, and 1,616,584 for samples with ≥2 ng/µL. QC pass rates were 88.6% (101/114), 98.4% (60/61), and 97.7% (429/439), respectively. Sample failures were concentrated below 1 ng/µL and particularly among samples recorded at zero concentration.

All 40 iCCA cases were successfully analyzed using OCA-P. *FGFR2* fusions were detected in 8 cases (20%), with patient ages ranging from 41 to 72 years (median: 52 years); 5 were male and 3 female. The *FGFR2::BICC1* fusion was the most frequent, identified in 4 of the 8 positive cases (50%). Notably, 4 of the *FGFR2*-positive cases had RNA yields below 2 ng/µL.

**Figure 3:**
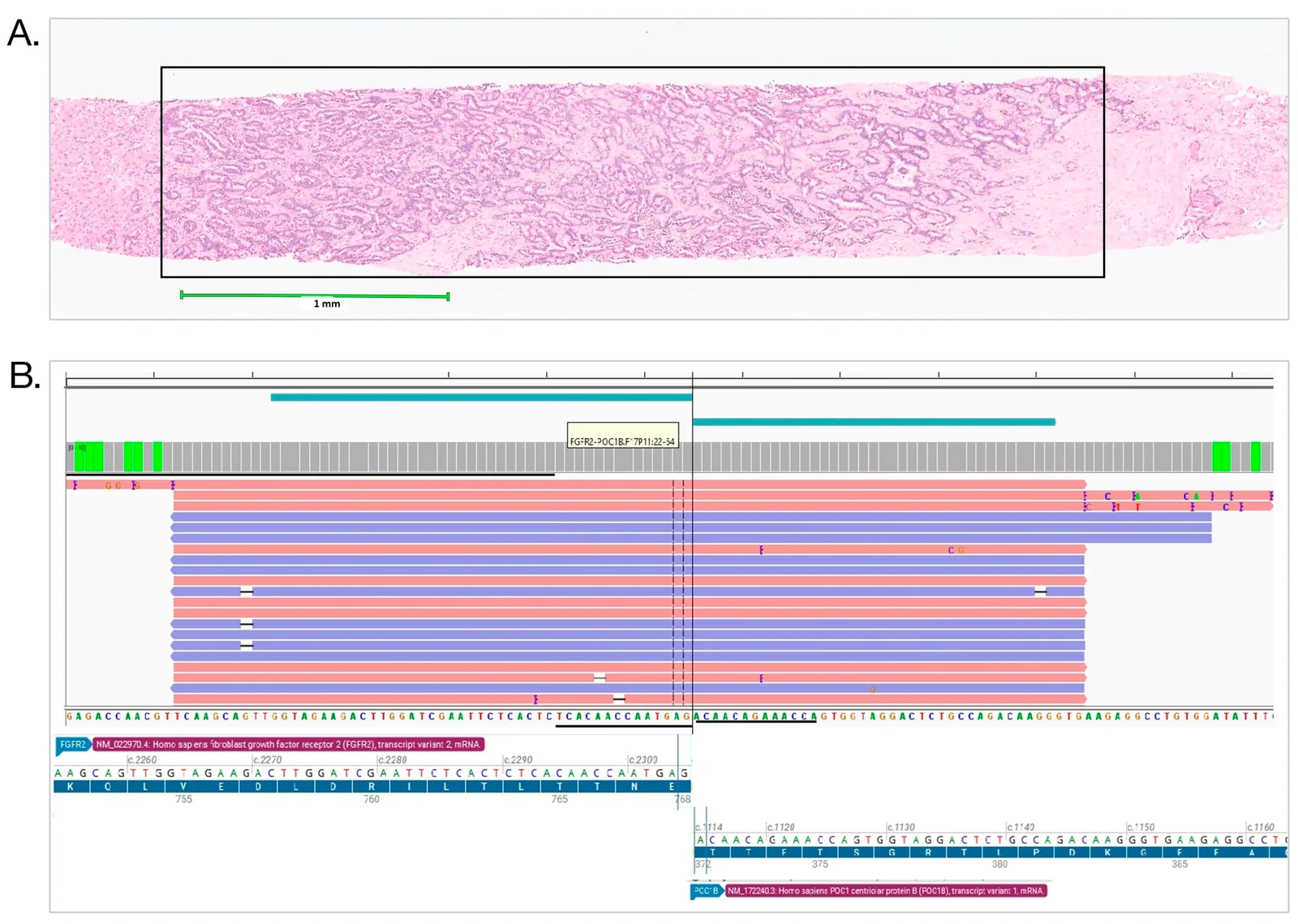
(**A**) H&E of liver core biopsy shows characteristic features of small duct type cholangiocarcinoma, indicated by a rectangle. There were 3 cores in this biopsy, the size of tumors measuring 3.5 mm, 3.7 mm and 4 mm, respectively. The tumor in (**A**) is 3.5 mm in size and from a single core. (**B**) IGV shows sequencing reads of in-frame *FGFR2::POC1B* fusion transcript.

No *FGFR2* fusions were identified in non-iCCA cases. A summary of the detected *FGFR2* fusion variants and corresponding RNA yields is provided in Table 1. One positive patient harboring *FGFR2- POC1B* fusion detected in a liver biopsy specimen with RNA yield of 1.6 ng/µL was referred to a clinical trial and showed clinical response to a FGFR2 inhibitor, although the trial center failed to detect that *FGFR2* fusion using a hybrid capture NGS assay (Figure 3). Statistical analysis using Fisher’s exact test revealed a significant association between *FGFR2* fusions and iCCA compared to non-iCCA cases (*p* = 0.01). Notably, RNA exon tiling, a 5′ to 3′ imbalance assay, failed to call the presence of gene fusions in 50% of *FGFR2* fusion-positive cases (Table 1). Case #8, however, was initially detected by RNA exon tiling and was subsequently sent to a reference laboratory (University Health Network, Toronto, ON, Canada), for a hybrid-capture assay (custom UHN CST Panel, version 1, on NextSeq instrument, using Illumina RNA Prep protocol and custom bioinformatics pipeline including STAR-Fusion and CTAT-SPLICING algorithms) which confirmed the presence of a novel *FGFR2* fusion [*FGFR2 (17)::C1orf50 (3)*] that has never been reported in the literature.

For DNA variants, *BAP1* variants were detected in 4 out of 8 *FGFR2* fusion-positive cases (Table 1), which is consistent with a prior study showing that *BAP1* variants are among the most common concomitant mutations found in cholangiocarcinoma harboring *FGFR2* fusions [22].

## 4. Discussion

While current guidelines emphasize the use of partner-agnostic RNA-based NGS platforms for *FGFR2* fusion detection in iCCA, our findings demonstrate that amplicon-based testing using OCA-P represents a viable and effective alternative in real-world diagnostic settings, particularly in centers where biopsy specimens constitute the majority of submitted material.

In our cohort, *FGFR2* fusions were identified in 20% of iCCA cases, consistent with, and slightly exceeding, previously reported prevalence rates of 10–16% [6,7]. Importantly, detection was achieved across a wide range of RNA input levels, including specimens with markedly limited nucleic acid yield.

A key strength of this study is the demonstrated robustness of the OCA-P assay in low-input and potentially degraded RNA samples. Notably, 50% of *FGFR2* fusion-positive cases exhibited RNA concentrations below 2 ng/µL, levels generally considered suboptimal for partner-agnostic sequencing platforms. Moreover, our workflow does not require RNA integrity assessment (e.g., RIN), further simplifying implementation in routine practice.

An illustrative case involved detection of an *FGFR2::POC1B* fusion in a biopsy specimen with an RNA concentration of 1.6 ng/µL. The patient was subsequently enrolled in a targeted therapy trial and demonstrated clinical response, despite failure of a partner-agnostic assay to detect the fusion. This highlights the clinical relevance of maintaining sensitive detection strategies for low-quality samples.

In comparison with published data, *FGFR2::BICC1* fusions accounted for 50% of detected rearrangements in our cohort, exceeding reported frequencies of approximately 25–30% [11,12,23]. This likely reflects the inherent limitation of amplicon-based panels in capturing noncanonical or rare fusion partners.

To demonstrate the utility of OCA-P in a real-world setting, it would be ideal in our study to test the samples by a hybrid-captured method parallelly. However, it is not feasible for our sample cohort, as 40% of our sample cohort (Figure 2) and among them, 50% of fusion-positive cases (Table 1), their RNA yields are considered suboptimal for a hybrid-captured method. In practice, these samples would be rejected for testing, as performing NGS on these suboptimal samples could result in false negative results, illustrated by the clinical case shown in Figure 3. The other 60% of samples with adequate RNA yield will require RNA quality assessment before testing, and we expect that a subset of these FFPE samples would not pass the required RNA quality. Given that a great number of cases in our study would be either suboptimal or ineligible for testing using a hybrid-captured method, the results of comparison between these two methods (i.e., OCA-P vs. a hybrid-captured method) could be inaccurate and misleading.

RNA exon tiling, a 5′-to-3′ imbalance assay implemented within an amplicon-based framework, is designed to infer the presence of novel fusion partners. However, in our evaluation, the RNA exon tiling approach failed to identify four cases harboring fusion genes (see Table 1), indicating that its sensitivity for detecting novel fusion partners remains suboptimal. Specificity could not be reliably assessed, as, in our experience, samples demonstrating positive exon tiling results were frequently not amenable to confirmation by partner-agnostic assays due to insufficient RNA yield. However, in our evaluation including samples from CBQA proficiency testing, RNA exon tiling was able to detect the presence of *FGFR2* fusions in two of the samples and confirmed by a reference method subsequently. As such, we would recommend pursing confirmation of positive calls of RNA exon tiling if the remaining RNA is sufficient for a hybrid-capture assay.

*FGFR2* fusion detection is complicated by extensive partner heterogeneity and variable genomic breakpoints, which challenge both assay design and bioinformatic analysis [17,18]. Consistent with this complexity, proficiency testing has revealed substantial inter-laboratory variability in detection performance. Failures could be linked to suboptimal analytical pipelines, particularly in partner-agnostic assays [18], highlighting the absence of a universally optimal NGS approach.

Against this backdrop and considering the additional constraints imposed by variable FFPE tissue quality and RNA yield, our findings support a complementary testing strategy that integrates amplicon-based and partner-agnostic methods. In real-world practice, implementing a triaged testing workflow based on RNA quantity and quality may enhance detection efficiency while maintaining an appropriate balance between sensitivity and resource utilization.

## 5. Conclusions

The OCA-P amplicon sequencing based assay reliably detected *FGFR2* fusion status in FFPE samples of intranhepatic cholangiocarcinoma. All 40 specimens were successfully analyzed, with *FGFR2* fusions being detected in 20% of cases, including several specimens with very low RNA yields (below 2 ng/µL). These results support the use of amplicon-based sequencing as a practical approach for specimens in which limited RNA quantity or quality may restrict the use of capture-based approaches. Importantly, however, confirmatory exon tiling approaches to detect *FGFR2* fusions should also be considered, but may be challenging in samples with poor RNA yields or quality. Limitations of this study include being a retrospective, single-center cohort with a relatively small sample size. Larger studies across multiple sites are therefore warranted. Given the breadth of different *FGFR2* fusion partners, the limitations of individual assay designs, and the difficulties inherent in RNA yield from FFPE biopsy specimens, a complementary testing strategy that incorporates both amplicon and fusion partner-independent sequencing assays may provide an effective means to maximize clinically relevant *FGFR2* fusion detection in routine practice.

## Figures and Tables

**Table 1:** Cases of intrahepatic cholangiocarcinoma harboring *FGFR2* fusion.

Case Number	Gender	Specimen Type	RNA Concentration (ng/µL)	RNA Variant	RNA Exon Tile	DNA Variant
1	Male	Liver, core biopsy	1.60	*FGFR2(17)::POC1B(11)*	No	N/A
2	Female	Liver, core biopsy	0.02	*FGFR2(17)::BICC1(3)*	No	N/A
3	Female	Liver, core biopsy	3.10	*FGFR2(17)::BICC1(3)*	No	N/A
4	Male	Liver, wedge resection	104.00	*FGFR2(17)::BICC1(3)*	No	*BAP1* c.379A > T VAF = 17%
5	Female	Liver, core biopsy	4.20	*FGFR2(17)::BICC1(3)*	Yes	*BAP1* *c.506A* > G VAF = 55%
6	Male	Abdominal wall nodule, core biopsy	0.80	*FGFR2(17)::TACC2(11)*	Yes	*BAP1* c.539T > A VAF = 49%
7	Male	Liver, core biopsy	1.40	*FGFR2 (17)::KCTD1 (2)*	Yes	*BAP1* c.418_427del VAF = 32%
8	Male	Liver core, biopsy	4.60	* *FGFR2 (17)::C1orf50 (3)*	Yes	*TP53* c.532delC VAF = 49%

* gene fusion detected by hybrid-capture assay.

## Data Availability

The data presented in this study are available on request from the corresponding author due to the privacy of personal genetic information.
